# 3,6-Dichloro-*N*-(4-fluoro­phen­yl)picolinamide

**DOI:** 10.1107/S1600536809024799

**Published:** 2009-07-04

**Authors:** Zhengde Tan, Yi Bing, Shen Fang, Zhao Kai, Yang Yan

**Affiliations:** a College of Chemistry and Chemical Engineering, Hunan Institute of Engineering, Xiangtan 411104, People’s Republic of China; b Guangxi Institute of Standards and Technology, Nanning 530022, People’s Republic of China

## Abstract

In the title compound, C_12_H_7_Cl_2_FN_2_O, the dihedral angle between the phenyl and pyridine rings is 42.5 (2) Å and an intramolecular N—H⋯N hydrogen bond occurs. The crystal structure is stabilized by C—H⋯O, C—H⋯F and C—Cl short contacts.

## Related literature

For the chemical and pharmacological properties of amides, see: Liu *et al.* (2005 ▶); Sladowska & Sieklucka-Dziuba (1999 ▶).
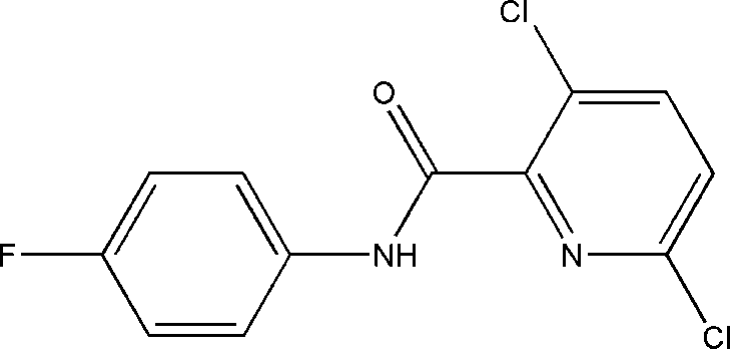


## Experimental

### 

#### Crystal data

C_12_H_7_Cl_2_FN_2_O
*M* *_r_* = 285.10
Orthorhombic, 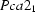

*a* = 24.921 (2) Å
*b* = 4.3735 (6) Å
*c* = 11.1723 (14) Å
*V* = 1217.7 (2) Å^3^
*Z* = 4
Mo *K*α radiation
μ = 0.53 mm^−1^
*T* = 298 K
0.45 × 0.33 × 0.31 mm

#### Data collection

Bruker SMART CCD diffractometer
Absorption correction: multi-scan (*SADABS*; Sheldrick, 1996 ▶) *T*_min_ = 0.796, *T*_max_ = 0.852
5652 measured reflections
1959 independent reflections
1582 reflections with *I* > 2σ(*I*)
*R*_int_ = 0.066

#### Refinement

*R*[*F* ^2^ > 2σ(*F* ^2^)] = 0.051
*wR*(*F* ^2^) = 0.140
*S* = 1.08
1959 reflections
163 parameters
1 restraint
H-atom parameters constrained
Δρ_max_ = 0.21 e Å^−3^
Δρ_min_ = −0.20 e Å^−3^
Absolute structure: Flack (1983 ▶), 826 Friedel pairs
Flack parameter: −0.04 (12)

### 

Data collection: *SMART* (Siemens, 1996 ▶); cell refinement: *SAINT* (Siemens, 1996 ▶); data reduction: *SAINT*; program(s) used to solve structure: *SHELXS97* (Sheldrick, 2008 ▶); program(s) used to refine structure: *SHELXL97* (Sheldrick, 2008 ▶); molecular graphics: *SHELXTL* (Sheldrick, 2008 ▶); software used to prepare material for publication: *SHELXTL*.

## Supplementary Material

Crystal structure: contains datablocks I, global. DOI: 10.1107/S1600536809024799/hg2522sup1.cif

Structure factors: contains datablocks I. DOI: 10.1107/S1600536809024799/hg2522Isup2.hkl

Additional supplementary materials:  crystallographic information; 3D view; checkCIF report

## Figures and Tables

**Table 1:** Hydrogen-bond geometry (Å, °)

*D*—H⋯*A*	*D*—H	H⋯*A*	*D*⋯*A*	*D*—H⋯*A*
N2—H2⋯N1	0.86	2.17	2.606 (5)	111

**Fig. 1.**
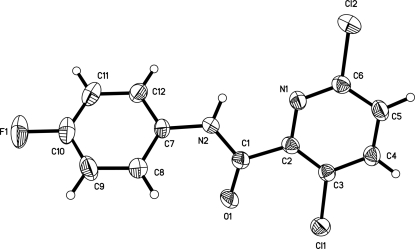
The molecular structure of the title compound showing 50% probability displacement ellipsoids for non-H atoms.

**Fig. 2.**
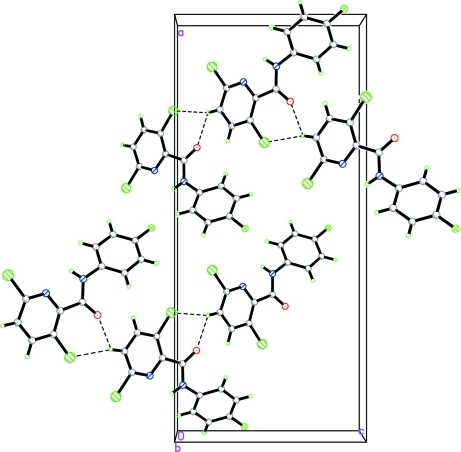
Crystal packing of the title compound, showing the hydrogen bonds as dashed lines

## supplementary crystallographic information

### Comment

The chemical and pharmacological properties of acid amides have investigated extensively, owing to their chelating ability with metal ions and to their potentially beneficial chemical and biological activties (Liu *et al.*,2005; Sladowska *et al.*, 1999). As part of our studies on the synthesis and characterization of these compounds, we report here the synthesis and crystal structure of 3,6-dichloro-*N*-(4-fluorophenyl)picolinamide. The C=O bond length is 1.200 (5) Å, indicating that the molecule is in the keto form. In the crystal structure, the molecules are stabilized by intramolecular N—H···N hydrogen bonds and C—H···O, C—H···F, C—Cl short contact.(Table 1 and Fig 2)

### Experimental

A solution of 3,6-dichloropicolinoyl chloride(10 mmol) in 50 ml toluene was added to a solution of 4-fluorobenzenamine (10 mmol) in 10 ml toluene. The reaction mixture was refluxed for 1 h with stirring then the resulting white precipitate was obtained by filtration, washed several times with ethanol and dried *in vacuo*(yield 90%). Elemental analysis calculated:*C*, 50.55; H, 2.47; N, 9.83%; found: C, 50.52; H, 2.49; N, 9.82%. Crystals were obtained by slow evaporation of a solution in methanol after one week.

### Refinement

H atoms were placed geometrically and refined using a riding model, with C—H=0.93 Å,*N*—H=0.86 Å, respectively, and *U*_iso_(H) = 1.2*U*_eq_(N) and *U*_iso_(H) = 1.2*U*_eq_(C).

### Crystal data

**Table:**

C_12_H_7_Cl_2_FN_2_O	*F*(000) = 576
*M**_r_* = 285.10	*D*_x_ = 1.555 Mg m^−^^3^
Orthorhombic, *P**c**a*2_1_	Mo *K*α radiation, λ = 0.71073 Å
Hall symbol: P 2c -2ac	Cell parameters from 1638 reflections
*a* = 24.921 (2) Å	θ = 2.9–27.0°
*b* = 4.3735 (6) Å	µ = 0.53 mm^−^^1^
*c* = 11.1723 (14) Å	*T* = 298 K
*V* = 1217.7 (2) Å^3^	Block, colorless
*Z* = 4	0.45 × 0.33 × 0.31 mm

### Data collection

**Table:**

Bruker SMART CCD diffractometer	1959 independent reflections
Radiation source: fine-focus sealed tube	1582 reflections with *I* > 2σ(*I*)
graphite	*R*_int_ = 0.066
φ and ω scans	θ_max_ = 25.0°, θ_min_ = 1.6°
Absorption correction: multi-scan (*SADABS*; Sheldrick, 1996)	*h* = −29→26
*T*_min_ = 0.796, *T*_max_ = 0.852	*k* = −5→5
5652 measured reflections	*l* = −13→11

### Refinement

**Table:**

Refinement on *F*^2^	Hydrogen site location: inferred from neighbouring sites
Least-squares matrix: full	H-atom parameters constrained
*R*[*F*^2^ > 2σ(*F*^2^)] = 0.051	*w* = 1/[σ^2^(*F*_o_^2^) + (0.0703*P*)^2^ + 0.2751*P*] where *P* = (*F*_o_^2^ + 2*F*_c_^2^)/3
*wR*(*F*^2^) = 0.140	(Δ/σ)_max_ < 0.001
*S* = 1.08	Δρ_max_ = 0.21 e Å^−^^3^
1959 reflections	Δρ_min_ = −0.20 e Å^−^^3^
163 parameters	Extinction correction: *SHELXL97* (Sheldrick, 2008)
1 restraint	Extinction coefficient: 0.064 (9)
Primary atom site location: structure-invariant direct methods	Absolute structure: Flack (1983), 826 Friedel pairs
Secondary atom site location: difference Fourier map	Flack parameter: −0.04 (12)

### Special details

**Table:**

Geometry. All e.s.d.'s (except the e.s.d. in the dihedral angle between two l.s. planes) are estimated using the full covariance matrix. The cell e.s.d.'s are taken into account individually in the estimation of e.s.d.'s in distances, angles and torsion angles; correlations between e.s.d.'s in cell parameters are only used when they are defined by crystal symmetry. An approximate (isotropic) treatment of cell e.s.d.'s is used for estimating e.s.d.'s involving l.s. planes.
Refinement. Refinement of *F*^2^ against ALL reflections. The weighted *R*-factor *wR* and goodness of fit *S* are based on *F*^2^, conventional *R*-factors *R* are based on *F*, with *F* set to zero for negative *F*^2^. The threshold expression of *F*^2^ > σ(*F*^2^) is used only for calculating *R*-factors(gt) *etc*. and is not relevant to the choice of reflections for refinement. *R*-factors based on *F*^2^ are statistically about twice as large as those based on *F*, and *R*- factors based on ALL data will be even larger.

### Fractional atomic coordinates and isotropic or equivalent isotropic displacement parameters (Å^2^)

**Table:**

	*x*	*y*	*z*	*U*_iso_*/*U*_eq_	
Cl1	0.29548 (4)	0.3691 (3)	0.97142 (12)	0.0657 (4)	
Cl2	0.10409 (7)	0.8942 (4)	0.69084 (13)	0.0830 (5)	
N1	0.14698 (14)	0.5722 (9)	0.8609 (3)	0.0479 (9)	
N2	0.11700 (14)	0.2081 (9)	1.0330 (3)	0.0468 (9)	
H2	0.0985	0.2817	0.9747	0.056*	
F1	−0.00073 (16)	−0.4220 (10)	1.3695 (4)	0.1088 (14)	
O1	0.20091 (13)	0.1700 (12)	1.1055 (4)	0.0917 (17)	
C1	0.17010 (17)	0.2639 (11)	1.0316 (4)	0.0491 (11)	
C2	0.18767 (17)	0.4557 (11)	0.9264 (4)	0.0449 (11)	
C3	0.24053 (16)	0.5164 (10)	0.8958 (4)	0.0430 (10)	
C4	0.2520 (2)	0.7023 (11)	0.7983 (4)	0.0523 (11)	
H4	0.2873	0.7439	0.7771	0.063*	
C5	0.2098 (2)	0.8244 (12)	0.7333 (5)	0.0566 (12)	
H5	0.2157	0.9518	0.6680	0.068*	
C6	0.15852 (18)	0.7482 (11)	0.7701 (4)	0.0493 (11)	
C7	0.08862 (17)	0.0410 (10)	1.1205 (4)	0.0418 (10)	
C8	0.1042 (2)	0.0484 (13)	1.2409 (5)	0.0595 (13)	
H8	0.1342	0.1585	1.2654	0.071*	
C9	0.0732 (2)	−0.1150 (15)	1.3226 (5)	0.0756 (17)	
H9	0.0829	−0.1168	1.4030	0.091*	
C10	0.0290 (2)	−0.2722 (14)	1.2864 (6)	0.0714 (16)	
C11	0.0144 (2)	−0.2826 (13)	1.1691 (6)	0.0688 (15)	
H11	−0.0155	−0.3947	1.1457	0.083*	
C12	0.04429 (18)	−0.1262 (13)	1.0851 (5)	0.0559 (13)	
H12	0.0346	−0.1332	1.0048	0.067*	

### Atomic displacement parameters (Å^2^)

**Table:**

	*U*^11^	*U*^22^	*U*^33^	*U*^12^	*U*^13^	*U*^23^
Cl1	0.0458 (6)	0.0924 (10)	0.0588 (7)	0.0066 (6)	0.0025 (6)	0.0119 (8)
Cl2	0.0762 (9)	0.0970 (11)	0.0756 (10)	−0.0019 (8)	−0.0244 (8)	0.0324 (9)
N1	0.052 (2)	0.050 (2)	0.042 (2)	−0.0034 (18)	0.0024 (18)	0.0056 (19)
N2	0.0447 (19)	0.055 (2)	0.040 (2)	−0.0029 (17)	−0.0053 (16)	0.0082 (18)
F1	0.110 (3)	0.108 (3)	0.108 (3)	−0.014 (2)	0.051 (2)	0.036 (2)
O1	0.048 (2)	0.148 (5)	0.079 (3)	0.003 (2)	−0.0076 (18)	0.068 (3)
C1	0.044 (2)	0.055 (3)	0.048 (3)	0.003 (2)	0.000 (2)	0.007 (2)
C2	0.047 (2)	0.055 (3)	0.032 (2)	0.005 (2)	0.0023 (18)	0.000 (2)
C3	0.049 (2)	0.043 (2)	0.037 (2)	−0.0003 (19)	0.0002 (19)	−0.0018 (18)
C4	0.056 (3)	0.058 (3)	0.043 (3)	−0.007 (2)	0.009 (2)	0.001 (2)
C5	0.071 (3)	0.057 (3)	0.042 (3)	−0.008 (2)	0.001 (2)	0.011 (2)
C6	0.054 (3)	0.052 (3)	0.042 (3)	−0.003 (2)	−0.006 (2)	0.001 (2)
C7	0.047 (2)	0.037 (2)	0.042 (3)	0.0010 (19)	0.009 (2)	0.0005 (19)
C8	0.054 (3)	0.073 (4)	0.052 (3)	−0.004 (3)	0.003 (2)	0.005 (3)
C9	0.082 (4)	0.096 (5)	0.049 (3)	0.008 (3)	0.017 (3)	0.018 (3)
C10	0.072 (4)	0.065 (4)	0.078 (4)	0.004 (3)	0.034 (3)	0.017 (3)
C11	0.053 (3)	0.062 (3)	0.092 (5)	−0.010 (2)	0.017 (3)	−0.001 (3)
C12	0.049 (3)	0.062 (3)	0.057 (3)	−0.007 (2)	0.003 (2)	−0.002 (2)

### Geometric parameters (Å, °)

**Table:**

Cl1—C3	1.734 (4)	C4—H4	0.9300
Cl2—C6	1.741 (5)	C5—C6	1.383 (6)
N1—C6	1.305 (6)	C5—H5	0.9300
N1—C2	1.351 (6)	C7—C12	1.383 (7)
N2—C1	1.346 (5)	C7—C8	1.400 (7)
N2—C7	1.411 (6)	C8—C9	1.393 (7)
N2—H2	0.8600	C8—H8	0.9300
F1—C10	1.356 (6)	C9—C10	1.361 (9)
O1—C1	1.200 (5)	C9—H9	0.9300
C1—C2	1.508 (6)	C10—C11	1.360 (9)
C2—C3	1.387 (6)	C11—C12	1.379 (8)
C3—C4	1.389 (6)	C11—H11	0.9300
C4—C5	1.385 (7)	C12—H12	0.9300
			
C6—N1—C2	118.6 (4)	N1—C6—Cl2	116.1 (3)
C1—N2—C7	126.5 (4)	C5—C6—Cl2	118.7 (4)
C1—N2—H2	116.7	C12—C7—C8	120.6 (4)
C7—N2—H2	116.7	C12—C7—N2	118.4 (4)
O1—C1—N2	124.0 (4)	C8—C7—N2	120.9 (4)
O1—C1—C2	122.7 (4)	C9—C8—C7	117.6 (5)
N2—C1—C2	113.3 (4)	C9—C8—H8	121.2
N1—C2—C3	120.5 (4)	C7—C8—H8	121.2
N1—C2—C1	114.5 (4)	C10—C9—C8	120.9 (5)
C3—C2—C1	125.1 (4)	C10—C9—H9	119.5
C2—C3—C4	120.1 (4)	C8—C9—H9	119.5
C2—C3—Cl1	124.0 (3)	F1—C10—C11	119.9 (6)
C4—C3—Cl1	115.9 (3)	F1—C10—C9	118.9 (6)
C5—C4—C3	118.7 (4)	C11—C10—C9	121.3 (5)
C5—C4—H4	120.6	C10—C11—C12	119.7 (5)
C3—C4—H4	120.6	C10—C11—H11	120.2
C6—C5—C4	116.9 (5)	C12—C11—H11	120.2
C6—C5—H5	121.6	C11—C12—C7	119.9 (5)
C4—C5—H5	121.6	C11—C12—H12	120.0
N1—C6—C5	125.2 (5)	C7—C12—H12	120.0
			
C7—N2—C1—O1	1.7 (8)	C2—N1—C6—Cl2	−179.2 (3)
C7—N2—C1—C2	−178.8 (4)	C4—C5—C6—N1	0.2 (8)
C6—N1—C2—C3	−1.6 (7)	C4—C5—C6—Cl2	−179.6 (4)
C6—N1—C2—C1	178.1 (4)	C1—N2—C7—C12	−147.3 (5)
O1—C1—C2—N1	−172.2 (5)	C1—N2—C7—C8	33.9 (7)
N2—C1—C2—N1	8.3 (6)	C12—C7—C8—C9	−0.7 (8)
O1—C1—C2—C3	7.5 (8)	N2—C7—C8—C9	178.1 (5)
N2—C1—C2—C3	−172.0 (4)	C7—C8—C9—C10	−0.9 (9)
N1—C2—C3—C4	1.1 (7)	C8—C9—C10—F1	−178.5 (5)
C1—C2—C3—C4	−178.5 (4)	C8—C9—C10—C11	2.0 (10)
N1—C2—C3—Cl1	−178.1 (3)	F1—C10—C11—C12	179.1 (5)
C1—C2—C3—Cl1	2.3 (7)	C9—C10—C11—C12	−1.4 (9)
C2—C3—C4—C5	0.1 (7)	C10—C11—C12—C7	−0.2 (8)
Cl1—C3—C4—C5	179.3 (4)	C8—C7—C12—C11	1.3 (8)
C3—C4—C5—C6	−0.7 (7)	N2—C7—C12—C11	−177.5 (4)
C2—N1—C6—C5	0.9 (7)		

### Hydrogen-bond geometry (Å, °)

**Table:**

*D*—H···*A*	*D*—H	H···*A*	*D*···*A*	*D*—H···*A*
N2—H2···N1	0.86	2.17	2.606 (5)	111
